# Population Structure and Genetic Diversity in the Natural Distribution of *Neolamarckia cadamba* in China

**DOI:** 10.3390/genes14040855

**Published:** 2023-03-31

**Authors:** Yan-Wen Lv, Zi-Han He, Yu Xiao, Kun-Xi Ouyang, Xi Wang, Xin-Sheng Hu

**Affiliations:** 1 College of Forestry and Landscape Architecture, South China Agricultural University, Guangzhou 510642, China; 2 Guangdong Key Laboratory for Innovative Development and Utilization of Forest Plant Germplasm, Guangzhou 510642, China

**Keywords:** *Neolamarckia cadamba*, nuclear ITS sequences, mitochondrial DNA, population structure, pollen flow, isolation by distance

## Abstract

*Neolamarckia cadamba* (Roxb.) Bosser is a fast-growing deciduous tree species and belongs to the *Neolamarckia* genus of the Rubiaceae family. This species has great economic and medical values in addition to being an important timber species for multiple industrial purposes. However, few studies have examined the genetic diversity and population structure in the natural distribution of this species in China. Here, we applied both the haploid nrDNA ITS (619 bp for aligned sequences) and mtDNA (2 polymorphic loci) markers to investigate 10 natural populations (239 individuals in total) that covered most of the distribution of the species in China. The results showed that the nucleotide diversity was π = 0.1185 ± 0.0242 for the nrDNA ITS markers and π = 0.00038 ± 0.00052 for the mtDNA markers. The haplotype diversity for the mtDNA markers was h = 0.1952 ± 0.2532. The population genetic differentiation was small ($F_{st\left(n\right)}$ = 0.0294) for the nrDNA ITS markers but large ($F_{st\left(m\right)}$ = 0.6765) for the mtDNA markers. There were no significant effects of isolation by distance (IBD), by elevation, and by two climatic factors (annual average precipitation and tem perature). A geographic structure among populations ($N_{st}<G_{st}$) was absent. Phylogenetic analysis showed a highly genetic mixture among individuals of the ten populations. Pollen flow was substantially greater than seed flow ($m_{p}/m_{s}\;\gg$ 1.0) and played a dominant role in shaping population genetic structure. The nrDNA ITS sequences were neutral and all local populations did not undergo demographic expansion. The overall results provide fundamental information for the genetic conservation and breeding of this miraculous tree.

## 1. Introduction

*N. cadamba* (2n = 44) is an evergreen deciduous and fast-growing tree species and belongs to the *Neolamarckia* genus of the Rubiaceae family [1,2]. This species is naturally distributed in Yunnan, Guangxi, and Guangdong provinces of South China and other subtropical and tropical regions, including Vietnam, Malaysia, Myanmar, India, Sri Lanka, and Australia [1,2]. *N. cadamba* can grow to 40–45 m in height and 100–160 cm in diameter [2]. It prefers high temperature and strong light habitats as well as relatively moist and fertile soil [2]. It is characterized by a rounded crown, straight trunk, and rapid growth. Its timber has the properties of straight texture, easy planning, fast drying, and hardness [3,4]. It is widely used for multiple industrial and commercial purposes, such as wood board, pulp, and paper making [5,6]. The species is also used as a material for woody forage to feed livestock [7], for nectar preparation, and for nutraceutical beverages [8,9]. In addition, the species has rich secondary metabolites in different tissues, such as cadambine, alkaloids, and triterpenoids, which have antioxidant, anti-inflammatory, antibacterial, and antimalarial effects [10,11,12,13,14]. Thus, this species is traditionally used as a medicinal plant to cure several diseases, such as diabetes, anemia, stomatitis, leprosy, cancer, infection, and other diseases [12]. Up to now, this “miraculous tree” species has been the subject of extensive provenance trials, clonal propagation, and plantations in South China [15,16].

Recent studies on genetic variation and breeding of *N. cadamba* covered wide aspects. Provenance trials in China were conducted to assess the genetic variation among provenances in height, DBH (the diameter at the breast height), and wood volume [15,16,17]. A progeny test was also reported in India [18] to estimate the genetic parameters of phenotypic traits and to select better individuals for genetic improvement. Modern biotechniques were applied to developing plant propagation and identifying functional genes, including tissue culture and propagation [19,20], transcriptome analysis of gene expression [21,22], expression sequence tags (ESTs) in xylem tissue [23], gene discovery of developing xylem tissue [4], the evolution of gene families [24], and polyploidization breeding [25]. A previous study used ISSR (inter-simple sequence repeat) dominant markers to analyze the genetic differentiation between two artificial populations and among six natural populations in Malaysia [26]. The results showed that 8.71% of the total genetic variation occurred between two artificial populations and 20.13% of the total genetic variation occurred among natural populations. Recently, Wang et al. [27] reported a complete sequence of the mitochondrial genome of this species and analyzed its phylogenetic relationships with other species of the Rubiaceae family. Zhao et al. [28] reported the sequences of nuclear genomes, which provided useful references for designing molecular markers for phylogenetic and population structure analyses. Nevertheless, population genetic structure and genetic diversity have not been investigated in China, which limits our understanding of a general picture of the genetic variation in the natural distribution of this species.

The ecological and evolutionary processes underlying the present population structure have not been examined as well. Although we could exclude the impacts of interspecific processes, such as incomplete lineage sorting and introgression/hybridization, other processes, such as seed/pollen flow and local adaptation, were likely involved in shaping population structure. Previous provenance trials showed that significant population differentiation occurred in growth traits [16], suggesting that selection participated in local population adaption. However, the provenance trials did not provide information on the effect of gene flow in shaping population structure. Given that *N. cadamba* is an anemophilous plant, pollen grains may be dispersed to long distances. Seeds are potentially locally distributed around mother trees due to gravity effects or dispersal by animals [29]. Thus, we hypothesized that pollen flow would dominantly contribute to the inter-population gene flow.

Using nuclear and organelle markers, we investigated population genetic structure and genetic diversity in the natural distribution of *N. cadamba* in China. Gene flow between populations could be inferred from the genetic structure analysis using molecular markers. For the nuclear genomes, we used the nuclear ribosomal internal transcribed spacer (nrDNA ITS) markers, which were frequently applied to studying phylogeography and population structure of both plant and animal species. For the organelle genomes, we used mitochondrial DNA (mtDNA) markers based on our previous sequencing of mitochondrial genomes of a few individuals where polymorphic loci were observed in some samples [27]. However, polymorphic loci were not found although several samples were tested according to the sequence of chloroplast DNA (cpDNA) [30]. Therefore, the mtDNA marker was used as the second marker in this study. It is well known that the nrDNA ITS markers are biparentally inherited, and their inter-population gene flow is achieved through seed and pollen flow. The mtDNA markers are maternally inherited and their inter-population gene flow is achieved through seed flow only. Combining the results from two types of markers helps to elucidate the relative effects of pollen versus seed flow on shaping population structure [31,32]. In addition, to infer historical dynamics, we examined if the existent natural populations underwent expansion after bottleneck effects. This would also provide additional background information for breeding and genetic conservation of this species in the future.

## 2. Materials and Methods

### 2.1. Population Sampling and DNA Extraction

**Figure 1:**
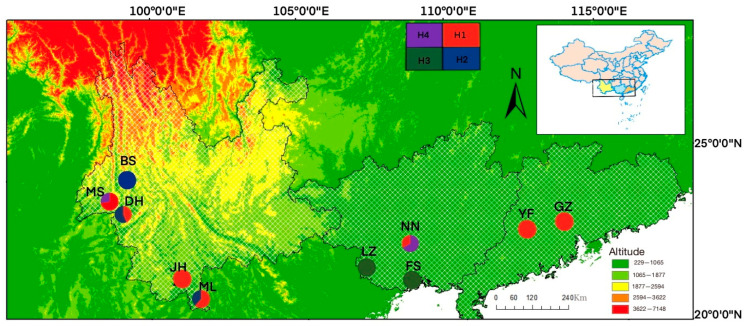
A map shows the ten sample sites of *N. cadamba* and the geographical distribution of the mtDNA haplotypes that were obtained from the concatenated sequences amplified by primers F1-R1 and F2-R2. The pie charts show the proportion of each haplotype in the ten populations. Each color in the pie chart represents one haplotype. H*i* (*i* = 1,2,3,4) represents the *i*th haplotype code.

Leaf samples were collected from ten natural populations (Table 1 and Figure 1), including three populations in Guangxi province (Longzhou, Fangchenggang, and Nanning), two populations in Guangdong province (Guangzhou and Yunfu), and five populations in Yunnan province (Baoshan, Dehong, Jinghong, Mangshi, and Mengla). These populations covered most of the natural distribution of *N. cadamba* in China. All sampling sites except for Baoshan were located at elevations below 1000 m. Trees were separated at least 50 m away in natural forest stands. A total of 239 individuals were analyzed in this study, with the sample size ranging from 15 to 32 among populations. DNA samples were extracted from silica-gel-dried leaves by following the CTAB 2X protocol [33]. The quality of DNA extraction was checked by 2% (*w*/*v*) agarose gel electrophoresis. All quantified DNA samples were stored at −20 °C for polymerase chain reaction (PCR) amplification.

### 2.2. Primer Screening, PCR Amplification, and Sequencing

For the mitochondrial genomes, we tried 11 pairs of primers that were selected from the relevant literature (Table S1), but their amplifications did not have single nucleotide polymorphisms (SNPs). We designed two pairs of primers from our previous sequencing of the mitochondrial genome of *N. cadamba* [27], which contained SNPs in their amplifications. These two pairs of primers were GAACATGGATTAGCATTATGTC/AT GCTAAGAGAGGGATGCTTCGC for the F1-R1 pair, and TTAGGGTCCG CTTACTTTG A/AACCGGGTAG ATGCTAAGAG for the F2-R2 pair.

For the nrDNA ITS markers, the forward and reverse primers were TCCTCCGCTTA TTGATATGC and GGAAGGAGAAGT CGTAACAAGG, respectively.

Each PCR amplification was conducted in a 25 μL reaction volume. The amplification volume included 1 μL of template DNA, 1.0 μL of each forward and reverse primer, 9.5 μL of ddH_2_O, and 12.5 μL of a mixed enzyme (0.1 U Tap polymerase, 500 μmol/L dNTPs, 20 mmol/LTris-HCl, 3 mmol/L MgCl_2_, and 100 mM KCl). The PCR amplification for nrDNA ITS and mtDNA primers is detailed below: (i) preheating at 95 °C for 4 min; (ii) annealing at a 50–65 °C temperature interval for 30 s (55 °C for ITS, 58 °C for F1-R1, and 53 °C for F2-R2); (iii) extension at 72 °C for 1 min, and repetition of the cycle of denaturation–annealing–extension for 35 times; (iv) 72 °C final extension for 10 min to make PCR amplification products be fully extended; and, finally, 4 °C to stop the reaction program. Then, the PCR products were run on 2% agarose gel electrophoresis. The results of gel running were examined by a gel imager, and the amplification products with clear and single bands were sent to Shanghai Biotech Biological Co., Ltd. (Shanghai, China) for sequencing. The Sanger method was used for sequencing. We checked all sequence data obtained from the company using Chromatogram Explorer 3.2 software. The sequences with high quality and without mixed peak signals were used for downstream analyses.

### 2.3. Analysis of Genetic Diversity

We aligned the sequences of both nrDNA ITS and mtDNA markers from 10 populations with MEGA7 [34] and removed those instable bases at the front and end. Two datasets were generated for analysis: one for the concatenated sequences from amplifications of primers F1-R1 and F2-R2 (Table S2) and the other for the nrDNA ITS sequences (Table S3).

DNAsp v5 [35] was used to estimate the haplotype diversity (h) of the mtDNA markers, the nucleotide diversity (π), and the effective population size-scaled mutation rate (θ) for both mtDNA and haploid nrDNA ITS markers [36,37,38]. The nucleotide diversity (π) refers to the pairwise nucleotide differences per nucleotide site [39]. The parameter θ per nucleotide site was estimated from the number of segregating sites among sequences in a sample [36]. TCS v1.21 [40] was used to map the evolutionary network among mitochondrial haplotypes.

### 2.4. Analysis of Population Genetic Structure

Population genetic structure was analyzed using DNAsp v5 [35] and Arlequin v3.0 [41] packages. Population differentiation was measured using $G_{st}$ [38], $F_{st}$ [42], $N_{st}$ [43,44], and $\phi_{st}$ [45]. The three indices ($G_{st}$, $\phi_{st}$, and $N_{st}$) have the same biological meaning as $\;F_{st}$ except that $G_{st}$ is used for the case of loci with more than two alleles and that $\phi_{st}$ and $N_{st}$ are estimated with haploid sequences. AMOVA (analysis of molecular variance) was performed using Arlequin v3.0 to estimate the distribution of variances within and between populations and the genetic differentiation coefficient ($\phi_{st})$. The geographical structure was inferred by testing if $N_{st}$ was significantly greater than $G_{st}$ [43].

Isolation by distance (IBD) was tested through the regression of $F_{st}/\left(1-F_{st}\right)$ on the logarithm of geographical distances [46],

(1) $$\frac{F_{st}}{1-F_{st}}=a+b\cdot \ln\left(\mathrm{geographic}\;\mathrm{distance}\right)$$

The geographical distance between two populations was calculated using their latitude and longitude coordinates (Table 1). A significant difference in the regression coefficient b from 0 means the presence of IBD effects. Pearson correlation between $F_{st}$ and geographical distance was also tested to check IBD effects in the case of many pairwise populations with complete differentiation ($F_{st}$ = 1) or without differentiation ($F_{st}$ = 0). In addition, isolation tests by elevation and by two climatic factors were performed. Mantel tests were conducted to test all types of isolation effects using the mantel function in R.

Phylogenetic relationships among individuals of the ten populations were analyzed using MEGA 7 [35]. The phylogenetic tree among populations was drawn using the ML (maximum likelihood) method [35]. Population structure was investigated using ADMIXTURE version 1.3.0 for the haploid nrDNA ITS sequences [47] and the results were plotted using the barplot function in R.

Gene flow was assessed under the assumption of the classical island model [48]. For maternally inherited mtDNA markers, population genetic differentiation, denoted by $F_{st\left(m\right)}$, is expressed by

(2) $$F_{st\left(m\right)}=\frac{1}{1+2N_{e}m_{s}}$$

where $N_{e}$ is the effective population size and $m_{s}$ is the migration rate of seeds. For the biparental nrDNA ITS markers, population genetic differentiation, denoted by $F_{st\left(n\right)}$, is expressed by

(3) $$F_{st\left(n\right)}=\frac{1}{1+2N_{e}\left(m_{s}+m_{p}/2\right)}$$

where $m_{p}$ is the migration rate of pollen [30,31]. From Ennos [30], the relative rate of pollen to seed flow is estimated by comparing $F_{st\left(m\right)}$ and $F_{st\left(n\right)}$,

(4) $$\frac{m_{p}}{m_{s}}=\frac{\left(1-F_{st\left(n\right)}\right)F_{st\left(m\right)}}{\left(1-F_{st\left(m\right)}\right)F_{st\left(n\right)}}-2$$

Standard deviation of the point estimate $m_{P}/m_{s}$ was estimated using the same method as Xiao [49]. The Jacknife method was used to estimate the variances in $F_{st\left(m\right)}$ and $F_{st\left(n\right)}$ [50], which were then used to calculate the standard deviation of $m_{P}/m_{s}$.

### 2.5. Analysis of Population Demography

We tested the neutrality of the nrDNA ITS markers using Tajima’s *D* [39] and Fu’s *F* statistics [51]. Tajima’s *D* was calculated as the normalized difference of π−θ. The neutral variation was implied when *D* was not significant from zero. This was the same case in testing neutrality using Fu’s *F* statistics. Significant negative values of Tajima’s *D* and Fu’s *F* also implied that a population had expanded after bottleneck effects. To further infer population demographic changes, mismatch distribution was analyzed using the Arlequin v3.0 package [41]. Under a hypothesis of population expansion after bottleneck effects, an unimodal distribution of the frequency of the observed number of pairwise different sites is expected, which fits for a single-peaked Poisson distribution. This analysis tested if the expected sum of square deviations (SSD) and the Harpending’s raggedness index (Rag) were greater than the observed SSD and Rag [52], respectively. Relevant parameters were $\theta_{0}$ = 2$N_{0}\mu$ and $\theta_{1}$ = 2$N_{1}\mu$, where $N_{0}$ and $N_{1}$ were the population sizes before and after population expansion, and $\tau \;\left(t\right)$ was the time elapsed since a sudden expansion.

## 3. Results

### 3.1. Genetic Diversity

The segments produced by the two primer pairs were about 650 bp for the primer F1-R1 amplification and about 400 bp for the primer F2-R2 amplification. Sequence alignment analysis confirmed that both amplified segments were located on genome 2 (GenBank Access No. MT364442) of Wang et al. [27]. We obtained 161 samples in total for the mtDNA marker (Table S2).

For the haploid nrDNA ITS markers, the PCR amplification size was about 750 bp. We obtained 239 samples of sequences for downstream analyses (Table S3).

**Figure 2:**
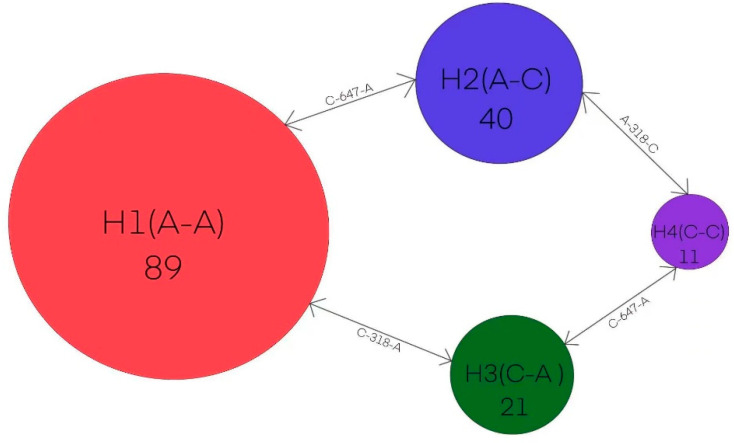
A network of mitochondrial haplotypes from the concatenated sequences of primers F1-R1 and F2-R2 amplifications. The circle sizes are proportional to the haplotype frequencies among 161 samples. The number of haplotypes is provided within each circle. Base substitutions between haplotypes are shown on the double-head arrows and the number of 318 or 647 on the double-head arrows refers to the position for base substitutions in the aligned sequences.

For the mtDNA markers, a total of 4 haplotypes were identified among the 161 sequenced individuals (Figure 2). Haplotype H1 (A-A) was the most frequent haplotype, accounting for 31% of all 161 samples, and occurred in 7 populations. This was followed by H2 (C-A) accounting for 28%, H3 (A-C) for 25%, and H4 (C-C) for 15% of all samples. Figure 1 shows the geographical distribution of these four haplotypes in the ten populations. Six populations were fixed by monomorphic haplotype (Table 2), including populations LZ, FS, GZ, YF, BS, and JH (h=0, π = 0). However, a high level of polymorphisms were observed in populations NN (h=0.5000, π = 0.0013), DH (h = 0.5140, π = 0.0007), MS (h = 0.4250, π = 0.0011), and ML (h=0.5130, π = 0.0007).

For the haploid nrDNA ITS markers, the nucleotide diversity ranged from π = 0.0806 in FS to 0.1616 in BS, with a mean of 0.1185 ± 0.0242 (Table 2). The effective population size-scaled mutation rate (θ) ranged from 0.0985 in YF to 0.1543 in LZ, with the mean of 0.1345 ± 0.0180. The θ estimates were slightly greater than the π estimates in all populations except for BS. All populations except for FS, YF, and DH had relatively large nucleotide diversity (π>0.1).

### 3.2. Population Genetic Structure

Analysis of molecular variance (AMOVA) indicated significant genetic differentiation among populations for both mtDNA and nrDNA ITS markers (Table 3). For the mtDNA markers, population differentiation coefficients were $\phi_{st}$ = 0.5533 at the locus amplified by the F1-R1 primers, 0.7955 by the F2-R2 primers, and 0.6755 for their concatenated sequences. For the nrDNA ITS markers, the population differentiation coefficient was $\phi_{st}$ = 0.0246. A major proportion of genetic variation occurred within populations, contrasting with the results of the analysis of the mtDNA markers.

A comparison of $N_{st}$ with $G_{st}$ showed that $N_{st}$ (=0.6764) was slightly smaller than $G_{st}$ (= 0.6893) for the mtDNA markers. This was the same case for the haploid nrDNA markers where $N_{st}$ (=0.0300) was slightly smaller than $G_{st}$ (=0.0340). The results indicated that the phylogeographic structure was absent in the spatial haplotype distribution (Figure 1 for the mtDNA markers only).

**Figure 3:**
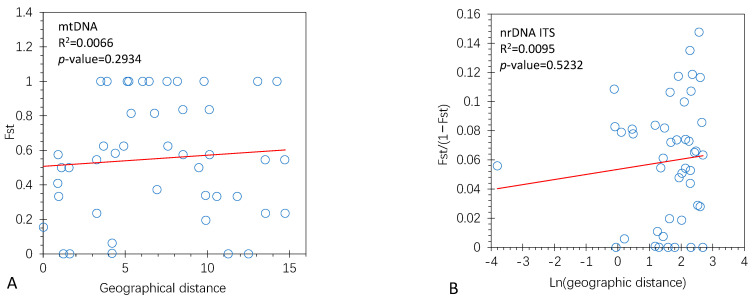
Effects of isolation by distance (IBD) on population genetic differentiation. (**A**) Pearson correlation between *F_st_* and geographic distance for the mtDNA markers. (**B**) The regression analysis of $F_{st}$/(1 −$\;F_{st}$) on geographic distance for the nrDNA ITS markers.

Figure 3 shows that the IBD effects were not significant for both types of markers. For the concatenated mtDNA markers (Figure 3A), Pearson’s correlation coefficient between $F_{st}$ and geographic distance was r = 0.0815, *p*-value = 0.2934. For the haploid nrDNA ITS markers, analysis of the regression of $F_{st}$/(1 − $F_{st}$) on the geographic distance was not significant, $F_{st}$/(1 − $F_{st}$) = 0.0534 + 0.0034 ln(geographical distance), $R^{2}$ = 0.0095, *p*-value = 0.5232. This correlation was also tested by the Mantel test and was not significant (Pearson’s correlation r = 0.0182, *p*-value = 0.32). Mantel tests also showed insignificant correlations between $F_{st}$ and the elevation difference (r = −0.0989, *p*-value = 0.896), between $F_{st}$ and the difference in annual average precipitation (r = 0.0582, *p*-value = 0.140), and between $F_{st}$ and the difference in annual average temperature (r = 0.0165, *p*-value = 0.336).

**Figure 4:**
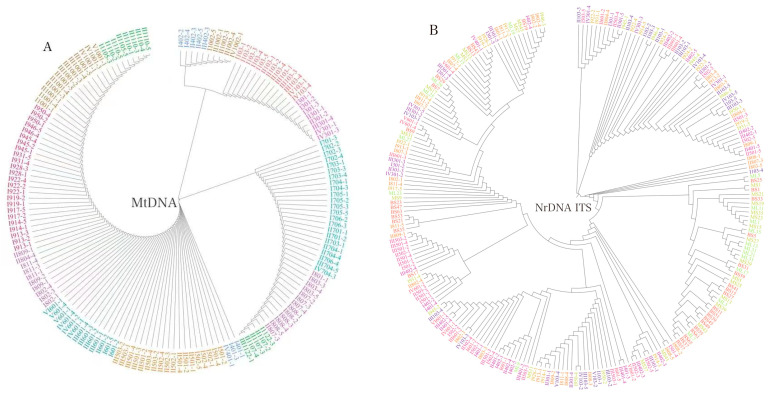
Phylogenetic relationships among individuals. (**A**) The mtDNA markers; (**B**) the nrDNA ITS markers. Individuals in different colors represent the origins of different populations.

**Figure 5:**
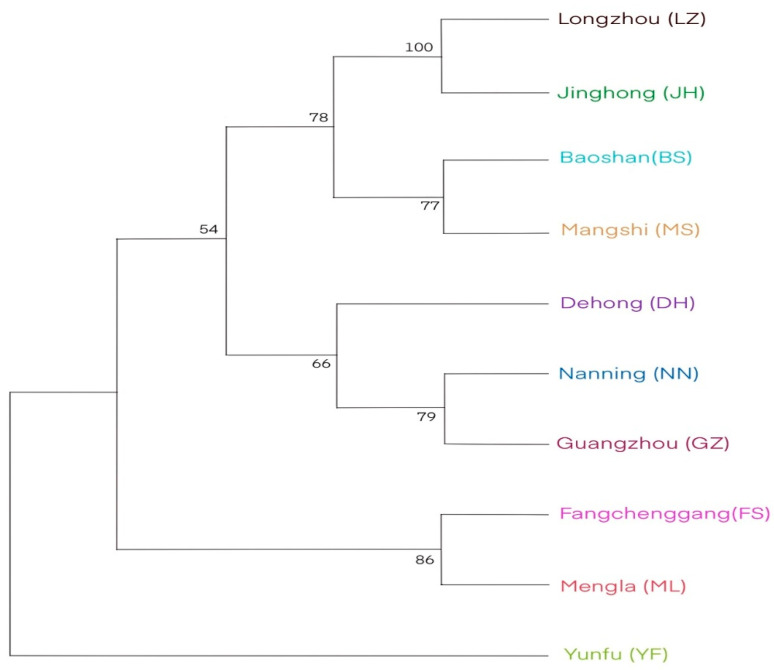
Phylogenetic relationships among ten populations of *N. cadamba* derived from the haploid nrDNA ITS markers. The phylogenetic tree was drawn using the ML method with 1000 bootstrap resamples.

**Figure 6:**
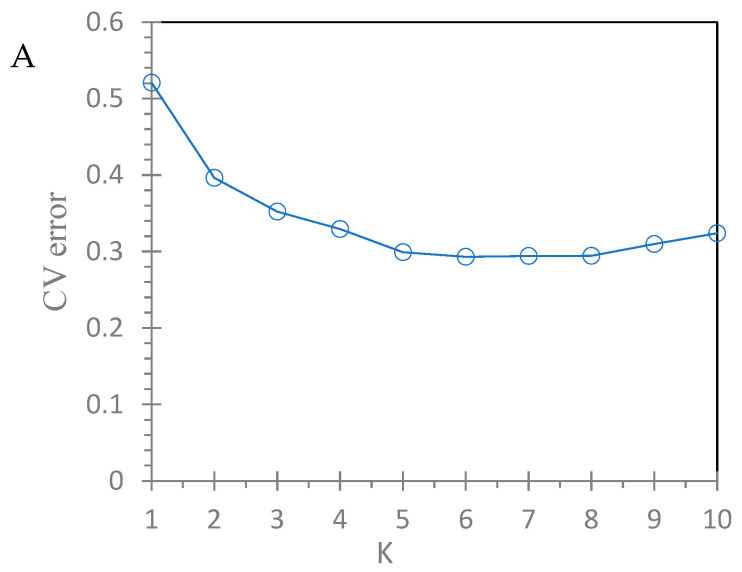

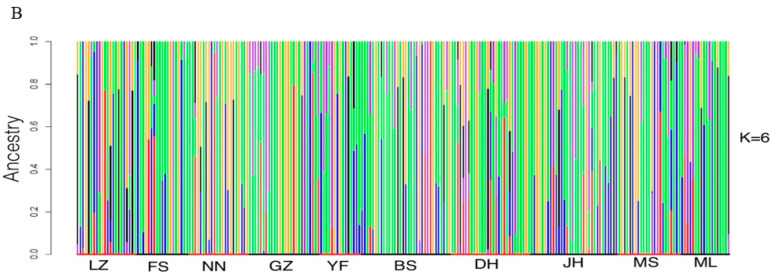
Population structure analysis of 239 individuals of *N. cadamba*. (**A**) Cross-validation (CV) errors for the number of subpopulations (*K*) changing from 1 to 10. (**B**) A partitioned map of each subpopulation with clustering assignments indicated in different colors.

Analysis of the phylogenetic relationship showed that individuals from the ten populations were genetically well mixed, indicating the presence of close genetic relationships among individuals using both the mtDNA (Figure 4A) and nrDNA ITS markers (Figure 4B). However, samples were genetically less mixed at the mtDNA loci than at the nrDNA ITS loci, consolidating that population genetic differentiation was relatively larger at the mtDNA loci. The phylogenetic relationship among populations did not match their geographical relationships (Figure 1 and Figure 5), consolidating the result of the absent geographical structure of genetic variation in the nrDNA ITS loci. Further analysis with ADMIXTURE showed that the optimal number of subpopulations was *K* = 6, with the minimum cross-validation (CV) error (CV error = 0.2993; Figure 6A). Figure 6B shows that individuals across populations were genetically well mixed by different proportions of the optimal six populations.

To estimate the standard deviation of $F_{st\left(m\right)}$, we used the two estimates of $\phi_{st}$ from AMOVA at the two polymorphic sites of the mtDNA markers. The mean estimates were 0.6765 ± 0.1712. The average and standard deviations of $F_{st\left(n\right)}$ estimated by the Jacknife method were 0.0294 and 0.0001, respectively. According to the method of Xiao [49], the ratio of pollen to seed flow was estimated, $m_{p}/m_{s}$ = 66.8440 ± 0.3351. Thus, pollen flow dominantly contributed to the gene flow among populations.

### 3.3. Population Demography

With the nrDNA ITS markers, Tajima’s *D* values were negative in all populations except for BS. The π estimate (0.1616) was slightly greater than the θ estimate (0.1532) in population BS. Fu’s *F* values were negative in five populations (LZ, FS, GZ, DH, and ML) but positive in the remaining five populations (NN, YF, BS, JH, and MS) (Table 2). All *p*-values were greater than 0.10, and the nrDNA ITS markers were neutral in all investigated populations. Populations with negative values of Tajima’s *D* and Fu’s *F* values potentially exhibited expansion after bottleneck effects.

Further analysis of mismatch distribution showed that a few local populations could expand after bottleneck effects. For instance, with population FS, the sum of squared deviation (SSD) was 0.093 (*p*-value = 0.0000) and Harpending’s raggedness index (Rag) was 0.136 (*p*-value = 0.0000). Other estimates of parameters were $\theta_{1}$ = 2$N_{1}\mu$ = 39.548 > $\theta_{0}$= 2$N_{0}\mu$ = 0.002, and the time elapsed since a sudden expansion episode τ = 2μt = 118.637. Population GZ did not undergo expansion. With population DH, we obtained SSD = 0.021 (*p*-value = 0.94), Rag = 0.027 (*p*-value = 0.10), $\theta_{1}$ = 43.77, $\theta_{0}$ = 0.004, and τ = 142.262. It possibly underwent an expansion after bottleneck effects to some extents. However, there was no a unimodal distribution for the frequency of the observed number of pairwise different sites in each population. Thus, we concluded that *N. cadamba* species potentially underwent a weak expansion in a few local regions but did not exhibit global expansion.

## 4. Discussion

### 4.1. Genetic Diversity

Although *N. cadamba* is a valuable species for multiple purposes, its molecular genetic variation has rarely been investigated in natural populations. We evaluated the genetic diversity in ten natural populations of *N. cadamba*. Both the nrDNA ITS and mtDNA markers were employed. These two types of markers were frequently applied to studying genetic diversity and population structure within and between species. The nrDNA ITS sequences were detected to be neutral from Tajima’s *D* tests and the results of isolation by climatic factors. The effects of deleterious mutation did not produce significantly negative values of Tajima’s *D* [39]. As expected, the overall level of nucleotide diversity was much lower for the mtDNA markers (π = 0.00038 ± 0.00052) than for the nrDNA ITS markers (π = 0.1185 ± 0.0242, θ = 0.1345 ± 0.0180) due to the lower mutation rate of the mitochondrial genome in plant species [53]. The haplotype diversity of the mtDNA markers was quite variable among populations, with the mean of h = 0.1952 ± 0.2532. A total of 6 populations (60%) were fixed by different haplotypes. There were absent phylogeographic structures ($G_{st}$ > $N_{st}$) in the spatial distribution of genetic diversity.

The overall nucleotide diversity with the nrDNA ITS markers in *N. cadamba* was greater than those found in many other plant species, such as *Forsythia suspensa* [54], *Hippophae rhamnoides* ssp. *sinensis* [55], *Toona ciliata* [49], *Primula obconica* [56], *Tamarix chinese* [57], *Achyranthes bidentata* [58], *Cycas revoluta* [59], *Spiraea alpina* [60], *Kandelia obovate* [61], and *Cerasus conradinae* [62]. However, the overall level of haplotype or nucleotide diversity with the mtDNA markers was smaller than those found in other angiosperms, such as *Cucurbita moschata* [63], *Medicago sativa* [64], *Fagus crenata* [65], *Cycas revoluta* [59], and *Pinus nigra* [66], but was comparable to that found in *T. ciliata* [49]. A previous study also indicated that higher percentages of polymorphic loci (45.3–74.6%) occurred using nuclear dominant ISSRs markers in three progeny trials of *N. cadamba* at the Landeh Nature Reserve, Semengok, and Sarawak of Malaysia [26]. Genetic diversity was high in the natural populations of *N. cadamba* in Malaysia.

### 4.2. Population Genetic Structure

As expected in theory [30,31], the observed patterns of the population genetic structure were contrasted for the maternally inherited mtDNA markers versus the biparentally inherited nrDNA ITS markers. For the nrDNA ITS markers, small but significant population genetic differentiation occurred, which accounted for about 2.94% of the total genetic variation. This result was in line with previous work on nuclear ISSRs marker variation in the natural populations of *N. cadamba* in Malaysia [5]. However, a major proportion of total genetic variation (more than 60%) occurred among populations for the mtDNA markers. These contrasting patterns could also be inferred from the phylogenetic relationships among individuals derived from the mtDNA markers versus the nrDNA ITS markers.

Two possible explanations could be responsible for such contrasting patterns. One explanation is related to the asymmetric dispersal between nuclear and mitochondrial genes. The fruits of *N. cadamba* are fleshy and spherical. Mature cones are about 6 cm in diameter [32]. Seeds are mainly locally distributed because the heavy cones are affected by gravity or are dispersed by animals. Most fallen seeds decay in the wet soil. Although mature cones are edible, cones are rarely directly consumed in China. Human activities could not effectively contribute to seed flow. However, the pollen of *N. cadamba* is windborne and can spread to long distances, especially in the small and scattered populations in South China where barriers to pollen flow are weak. The nrDNA lTS markers may be dispersed through both pollen and seed flow. The mtDNA markers are dispersed through seed flow only. The spatial distribution of mtDNA haplotypes implies restrictive seed flow among populations despite absent IBD effects. Therefore, these differences yielded the contrasting patterns of population structure.

The second explanation is related to the reproductive ecology of *N. cadamba* although its mating system has not been scored. The mode of the reproductive system influences pollen dispersal and species range [67,68]. The high genetic diversity but small population genetic differentiation, observed from analysis of the neutral nrDNA ITS markers, imply that *N. cadamba* is potentially predominantly outcrossing, which facilitates pollen flow. Our estimate of the relative rate of pollen to seed flow supported the hypothesis that pollen flow played a dominant role in shaping the population genetic structure of *N. cadamba*. Compared with other tropical plants, such as *Laelia rubescens* ($m_{p}/m_{s}$ = 13.67) [69], *Dactylorhiza umbrosa* (8.4–12.01) [70], and *T. ciliata* (1.3741 at the species level) [49], *N. cadamba* more heavily relies on pollen dispersal to shape population structure.

### 4.3. Implications for Genetic Resource Management

Two implications could be gained from the findings of this study. One implication is concerned with genetic conservation since population structure provides fundamental information for managing genetic resources [71]. Although genetic conservation is currently not a major issue in this species, it could be potentially important in the future [26]. Because of its economic and medical value, *N. cadamba* has been overharvested and, therefore, the natural population density is declining due to its narrow distribution in South China. Our results implicate that most local populations did not undergo demographic changes. Thus, on the evidence of small population differentiation, it would be appropriate to focus on a few populations in genetic conservation.

The second implication is concerned with breeding that is currently being conducted [16,17]. Although small population differentiation was observed with the neutral nrDNA ITS markers, the small population differentiation could also occur for some adaptive quantitative traits. Previous provenance trials showed that significant but small differences were present among provenances in tree height, DBH, and wood volume [17]. From the results of both population structure and provenance trials, it is speculated that provenance selection could be effective only for the adaptive traits but not for the neutral or nearly neutral traits. However, the genetic gain from provenance selection could be small, given the extensive pollen flow that effectively reduced population genetic differentiation. Therefore, artificial selection should concentrate on selection from families within provenances, or selection from individuals within families within provenances in breeding [72,73].

## 5. Conclusions

*N. cadamba* is a fast-growing timber tree species in South China. The species, also known as a miraculous tree, is exploited as a medicinal plant in addition to its use for industrial purposes. Here, we used both the mtDNA and nrDNA ITS markers to investigate ten populations that covered most of the natural distribution of *N. cadamba* in South China. Genetic diversity for both types of markers was randomly distributed in space, without phylogeographic structure. The genetic diversity was high from the analysis of the nrDNA ITS markers but low from the analysis of the mtDNA markers. Population genetic variation was mainly distributed within populations for the nrDNA ITS markers but among populations for the mtDNA markers. Effects of isolation by distance were absent among populations. The phylogenetic analysis showed close genetic relationships among individuals of the ten populations or among populations. The relative rate of pollen to seed flow was much greater than one, implying that pollen flow played a dominant role in contributing to gene flow of *N. cadamba*. Populations had not experienced expansion events and the nrDNA ITS sequences were selectively neutral. The overall results provide fundamental information for genetic conservation and breeding of *N. cadamba* in South China.

## Figures and Tables

**Table 1:** Locations, sample sizes, and two climatic factors of ten populations of *N. cadamba* from China.

Location	Code	Sample size	Longitude (E)	Latitude (N)	Elevation (m)	AAP # (mm)	AAT # (°C)
Longzhou, Guangxi	LZ	22	106.86	22.35	269	1260	22.2
Fangchenggang, Guangxi	FS	19	108.35	21.70	235	2512	21.8
Nanning, Guangxi	NN	22	108.37	22.82	80	1304.2	21.7
Guangzhou, Guangdong	GZ	26	113.27	23.13	10	1696.5	22.1
Yunfu, Guangdong	YF	15	112.05	22.92	346	1670.5	21.5
Baoshan, Yunnan	BS	33	99.17	25.14	1670	1710	17.4
Dehong, Yunnan	DH	29	98.61	24.44	780	1544	18.9
Jinghong, Yunnan	JH	32	100.81	22.03	552	1197	21.0
Mangshi, Yunnan	MS	23	98.59	24.43	913	1650	19.6
Mengla, Yunnan	ML	18	101.57	21.46	631	1540	21.0

#: AAP, annual average precipitation; AAT, annual average temperature.

**Table 2:** Genetic diversity of mtDNA and nrDNA ITS markers and the neutrality tests of the haploid nrDNA ITS sequences in ten populations of *N. cadamba*.

Population	mtDNA Marker		Haploid nrDNA ITS		Tajima’s D #	Fu’s F #
	*h*	*π*	*π*	*θ*		
LZ	0.0000	0.0000	0.1313	0.1543	−0.6107	−0.4676
FS	0.0000	0.0000	0.0806	0.1101	−1.1253	−1.0625
NN	0.5000	0.0013	0.1311	0.1416	−0.3029	0.1430
GZ	0.0000	0.0000	0.1128	0.1397	−0.7651	−0.1203
YF	0.0000	0.0000	0.0969	0.0985	−0.0728	0.2672
BS	0.0000	0.0000	0.1616	0.1532	0.0204	−0.0730
DH	0.5140	0.0007	0.0921	0.1313	−1.1649	−0.8674
JH	0.0000	0.0000	0.1391	0.1391	−0.4845	0.0783
MS	0.4250	0.0011	0.1229	0.1469	−0.6528	0.0964
ML	0.5130	0.0007	0.1164	0.1298	−0.4206	−0.0352

#: *p*-values for all Tajima’s *D* and Fu’s *F* tests were greater than 0.1—not significant.

**Table 3:** Analysis of molecular variance (AMOVA) of both mtDNA and nrDNA ITS markers of *N. cadamba*.

Marker	Source of Variation	d.f.	Sum of Square	Variance Component	Percentage of Variance (%)	*ϕ_st_*	*p*-Value
mtDNA *F1-R1* and *F2-R2*	Among populations	9	40.533	0.2750	67.55	0.6755	0.00
	Within populations	151	19.951	0.1321	32.45		
	Total	160	60.484	0.4071			
nrDNA ITS	Among populations	9	542.301	0.9507	2.46	0.0246	0.0009
	Within populations	229	8628.302	37.6782	97.54		
	Total	238	9170.603	38.6289			

## Data Availability

All data sets used in this study are provided in Tables S2 and S3 in the Supplementary Materials.

## Supplementary Materials

The following supporting information can be downloaded at: https://www.mdpi.com/article/10.3390/genes14040855/s1, Table S1: Dataset of 13 pairs of mitochondrial DNA primers tested in *N. cadamba*; Table S2: Dataset of 161 samples of mitochondrial sequences of *N. cadamba*. Each sample was a concatenated sequence of F1-R1 and F2-R2 amplified segments; Table S3: Dataset of 239 samples of nrDNA ITS alignment sequences of *N. cadamba*.
