# Supplementary material for: Population Structure and Genetic Diversity in the Natural Distribution of Neolamarckia cadamba in China
Source: Genes (Basel). 2023 Mar 31;14(4):855. doi: 10.3390/genes14040855 (PMC10137889; doi:10.3390/genes14040855)
Supplement: Supplementary file 1 [file genes-14-00855-s001.zip › genes-2253822-supplementary.pdf]

Supplementary Materials

Table S1. Thirteen pairs of primers from mitochondrial DNA tested in *Neolamarckia cadamba*

Primer pair	Forward and reverse primer sequences	Annealing temperature (°C)
cox3f/r	CCGTAGGAGGTGTGATGT<br>CTCCCCACCAATAGATAGAG	55
nad2-1/2-2	AATGTGGGTTGGCTCAG<br>TGGTTATTAGATACCGAAG	62
orf25/nad4L	CTGTYTTTTCGCACTTAGGC<br>GTCCGRGGTACTATTGCTGT	55
nad6f/r	TGAGTGGGTCWGTCGTCCTC<br>TGATACTTTCTGTTTTGTCG	58
nad9f/r	GGTCATCTCAATGGGYTCAG<br>TATAGTTGGGAGACTTTACC	52
orf25f/r	AAGACCRCCAAGCYYTCTCG<br>TTGCTGCTATTCTATCTATT	50
rps12/nad3	TTTCTTCTCTACCATGACGA<br>TGATCCYACTCGGTSTTCCT	55
rps4f/r	CSTTTCYGCTCCGAAGAG<br>TCTCCGAAGATTGAGG	58
nad7-1/7-2	ACCTCAACATCCTGCTGCTC<br>CGATCAGAATAAGGTAAAGC	47
nad7-2/7-3	GCTTTACCTTATTCTGATCG<br>TGTTCTTGGGCCATCATAGA	57
nad1-2/1-3	GCATTACGATCTGCAGCTCA<br>GGAAGCCGATTAGTTTCTGC	56
F1/R1	GAACATGGATTAGCATTATGTC<br>ATGCTAAGAGAGGGATGCTTCGC	58
F2/R2	TATAGGGTCCGCTTACTTTGA<br>AACCGGGTAAGATGCTAAGAG	53

Table S2: One hundred and sixty-one samples of mitochondrial sequences of *N. cadamba*. Each sample was a concatenated sequence of F1-R1 and F2-R2 segments.

Table S3: Two hundred and thirty-nine samples of nrDNA ITS alignment sequences of *N. cadamba*
